# A hybrid FeOx/CoOx/Pt ternary nanocatalyst for augmented catalysis of formic acid electro-oxidation

**DOI:** 10.1038/s41598-024-67834-9

**Published:** 2024-08-05

**Authors:** Ahmad M. Mohammad, Bilquis Ali Al-Qodami, Islam M. Al-Akraa, Nageh K. Allam, Hafsa H. Alalawy

**Affiliations:** 1 Chemistry Department, Faculty of Science, Cairo University, Cairo, 12613 Egypt; 2 Chemistry Department, Faculty of Education and Applied Science, Hajjah University, Hajjah, Yemen; 3 Department of Chemical Engineering, Faculty of Engineering, The British University in Egypt, Cairo, 11837 Egypt; 4 Energy Materials Laboratory, School of Sciences and Engineering, The American University in Cairo, New Cairo, 11835 Egypt

**Keywords:** Iron oxide, Cobalt oxide, Platinum, Formic acid oxidation, CO tolerance, Liquid Fuel cells, Catalysis, Electrochemistry, Energy, Materials chemistry

## Abstract

Platinum-based catalysts that have long been used as the anodes for the formic acid electro-oxidation (FAO) in the direct formic acid fuel cells (DFAFCs) were susceptible to retrogradation in performance due to CO poisoning that impaired the technology transfer in industry. This work is designed to overcome this challenge by amending the Pt surface sequentially with nanosized cobalt (nano-CoOx, fibril texture of ca. 200 nm in particle size) and iron (nano-FeOx, nanorods of particle size and length of 80 and 253 nm, respectively) oxides. This enriched the Pt surface with oxygenated groups that boosted FAO and mitigated the CO poisoning. The unfilled d-orbitals of the transition metals and their tendency to vary their oxidations states presumed their participation in a faster mechanism of FAO. Engineering the Pt surface in this FeOx/CoOx/Pt hierarchy resulted in a remarkable activity toward FAO, that exceeded four times that of the Pt catalyst with up to ca. 2.5 times improvement in the catalytic tolerance against CO poisoning. This associated a ca. − 32 mV shift in the onset potential of FAO which increased to − 40 mV with a post-activation of the same catalyst at − 0.5  in 0.2 mol L^–1^ NaOH, displaying the catalyst's competitiveness in reducing overpotentials in DFAFCs. It also exhibited a favorable amelioration in the catalytic durability in long-termed chronoamperometric electrolysis. The electrochemical impedance spectroscopy and the CO stripping voltammetry were employed to elucidate the origin of enhancement.

## Introduction

The energy and environmental crises have become global issues as a result of the overexploitation and rapid depletion of fossil fuels^1,2^. Currently, H_2_ is enjoying unprecedented momentum as a lively clean fuel and energy carrier for its abundance, small size, odorless, colorless, tasteless, no carbon content and high mass energy density (143 MJ kg^−1^)^3^. Hydrogen fuel cells (HFCs) that resemble the galvanic reactors in converting the chemical energy into electricity utilizing the H_2_ (that can be supplied directly or from proton-containing or storing materials) oxidation and O_2_ reduction have found potential applications in transportation and in several portable, stationary and emergency backup power devices^4–6^. Yet, H_2_ owns a low volumetric energy density (~ 10 kJ L^−1^ at 298 K, 1 atm), extreme flammability, very low density in the gaseous state, high cost of miniaturization for its containers and insecure delivery, that stood obstacles against a practical commercialization^3,7–9^. Suggested scenarios to overcome these challenges included the use of various solid and liquid hydrogen storage materials as metal hydrides^10^, sodium borohydride^11^, hydrazine hydrate^12^, ammonia^13^ and amine-borane complexes^14^. However, their exorbitant price and their poisonous, flammable and explosive nature have limited their implementation^7^.

Formic acid (FA) has next appeared as a potential hydrogen carrier with a high availability, non-toxicity, non-flammability, high hydrogen capacity (~ 4.4% by mass), high volumetric hydrogen content (53 g L^−1^ under standard temperature and pressure), high specific energy (5.3 MJ kg^−1^) and high volumetric energy density (6.4 MJ L^−1^ at ambient conditions)^9,15–20^. It compensates its comparatively lower theoretical energy density to that (~ 22 MJ kg^−1^) of methanol with its much lower crossover rate through the Nafion membrane; opening opportunity to use much higher fuel concentrations and to design portable compact power systems^21–25^. However, to efficiently comply FA for fuel cells' technology and to adopt the direct formic acid fuel cells (DFAFCs) for a convenient, economic and robust transformation of the chemical energy into electricity, durable and competent catalysts for the FA electro-oxidation (FAO) must be developed.

Platinum and Pt-based materials have long been recommended for the oxidation of small organic molecules including FA^20,26,27^. However, the high price of Pt as a precious element together with its deactivation in a long-termed operation by accumulated poisonous intermediates (e.g., CO) remained an issue to be resolved^28,29^. To design cost-effective catalysts and to improve the electrocatalysis of FAO in DFAFCs, it is critical to understand the FAO mechanism and the variables affecting the reaction kinetics^30^. Since the 70s, several mechanisms have been proposed for the FAO on Pt, which have recently been reevaluated after several spectroscopic studies and density functional theory (DFT)-based theoretical calculations^26^. Capon and Parsons^31^ suggested the first possible mechanism, consisting of the direct and indirect routes. A fast reaction through a reactive intermediate, which is immediately oxidized further to CO_2_, is involved in the direct route. The ^–^COOH species (bonded by a carbon atom) was previously suggested as a potential candidate, but no spectroscopic evidence could be obtained in this direction that could be replicated. Formate (HCOO^–^) was proposed by Osawa et al.^32,33^ as a reactive intermediate in the direct route in a bridge-bonded configuration, which means both oxygen atoms are bonded to two surface sites. The other indirect route involves a step in the creation of an inhibiting intermediate that impedes further FA adsorption. The adsorbed CO (CO_ads_) was established unambiguously as the poisonous species for this indirect route^26,34^. However, the mechanism of the dual route and the role of the bridge-bonded formate as an active intermediate were discussed controversially^35^. One significant argument is that the faradaic current observed experimentally increases more rapidly than the coverage of the bridge-bonded formate in chronoamperometric measurements with different concentrations of FA. This means that in the dominant reaction route, the adsorbed bridge-bonded formate cannot be regarded as a reaction intermediate. Others proposed a third route for the FAO mechanism, based on the fact that the formate is a spectator rather than a reactive intermediate^36–38^. However, studies using improved infrared techniques which allowed monitoring the reactions dynamics together with DFT calculations showed that formate could be an active intermediate in the FAO^39,40^. While CO_ads_ initial formation rate is very slow, its accumulation on the Pt surface seriously poisons the active sites and impedes the direct route. The low reserve and high price of Pt restrict its commercial applications; thus, it is necessary to design and develop high effective Pt-based catalysts with high utilization that could reduce the use of Pt while improving the catalytic activity for FAO^41^. Modifying the Pt surface with metal (e.g., Sn^42^, Bi^43^, Au^44–46^, Ni^47^, Co^48^, and Fe^49^) and/or transition metal oxides ( TMOs, e.g., NiOx^50^, CoOx^21,51^, WO_3_^52^, MnOx^53–55^ and FeOx^56–59^) could effectively minimize and possibly eliminate this poisoning with a significant geometrical (third-body), *bi*-functional and/or electronic improvement of the catalytic FAO. According to the Langmuir–Hinshelwood model, poisoning CO species can be oxidatively desorbed from the Pt surface after being hydroxylated at high overpotentials in aqueous solutions ^60^. This desorption was accelerated at earlier overpotentials when the Pt surface was modulated with CoOx or FeOx, that promoted the electrochemical dissociation of H_2_O at the Pt surface^61,62^. The existence of TMOs (having a tendency to convert easily between low and high oxidation states, and having unfilled d-orbitals) at the Pt surface is expected, moreover, to accommodate the transferred electrons during FAO in a faster reaction mechanism, that in turns, speeds up the oxidation kinetics. Recently, Pt-based ternary catalysts have emerged as promising candidates compared to bimetallic alloys because of their ability to enhance the structural properties of Pt and thus maintaining its catalytic activity for long durations^61^. In this study, a ternary FeOx/CoOx/Pt catalyst is designed; aiming at increasing its catalytic activity and CO tolerance toward the FAO.

## Experimental

### Materials and measurements

Ferrous sulfate heptahydrate (FeSO_4_.7H_2_O), cobalt (II) sulfate (CoSO_4_), formic acid (HCOOH), sodium hydroxide (NaOH), sulfuric acid (H_2_SO_4_) and sodium sulfate (Na_2_SO_4_) were purchased from Riedel–de Haen, Sigma Aldrich and Merck with high purity and analytical grade. The electrochemical measurements were carried out in a three-electrode electrochemical cell with the aid of EG&G potentiostat (model 273) operated with Research Electrochemistry software (ECHEM M270, version 4.3 for Windows). A Pt electrode (*d* = 3.0 mm), spiral platinum wire and Ag/AgCl/KCl (sat.) were used as the working, counter and reference electrodes, respectively. All potentials will then be measured in reference to the Ag/AgCl/KCl (sat.) electrode. All measurements were carried out at room temperature (~ 25 ± 1 °C). The electrochemical characterization and the electrocatalytic activity measurements of the various catalysts were examined by cyclic voltammetry (CV) and electrochemical impedance spectroscopy (EIS) in 0.3 mol L^−1^ FA (pH = 3.5) solution. The morphology of the catalyst was inspected with field-emission scanning electron microscopy (FE-SEM, Zeiss Ultra 60) at an accelerated voltage of 8 kV and a working distance of 2.8–3.2 mm. Whereas, the energy dispersive X-ray spectroscope (EDX) was used to determine the composition of the various catalysts. The crystal structures of the catalysts were evaluated using Bruker D8-Discover diffractometer having Cu K radiation source with a wavelength of 1.54 Å, operated at 40 kV, and 40 mA, Germany.

**Figure 1:**
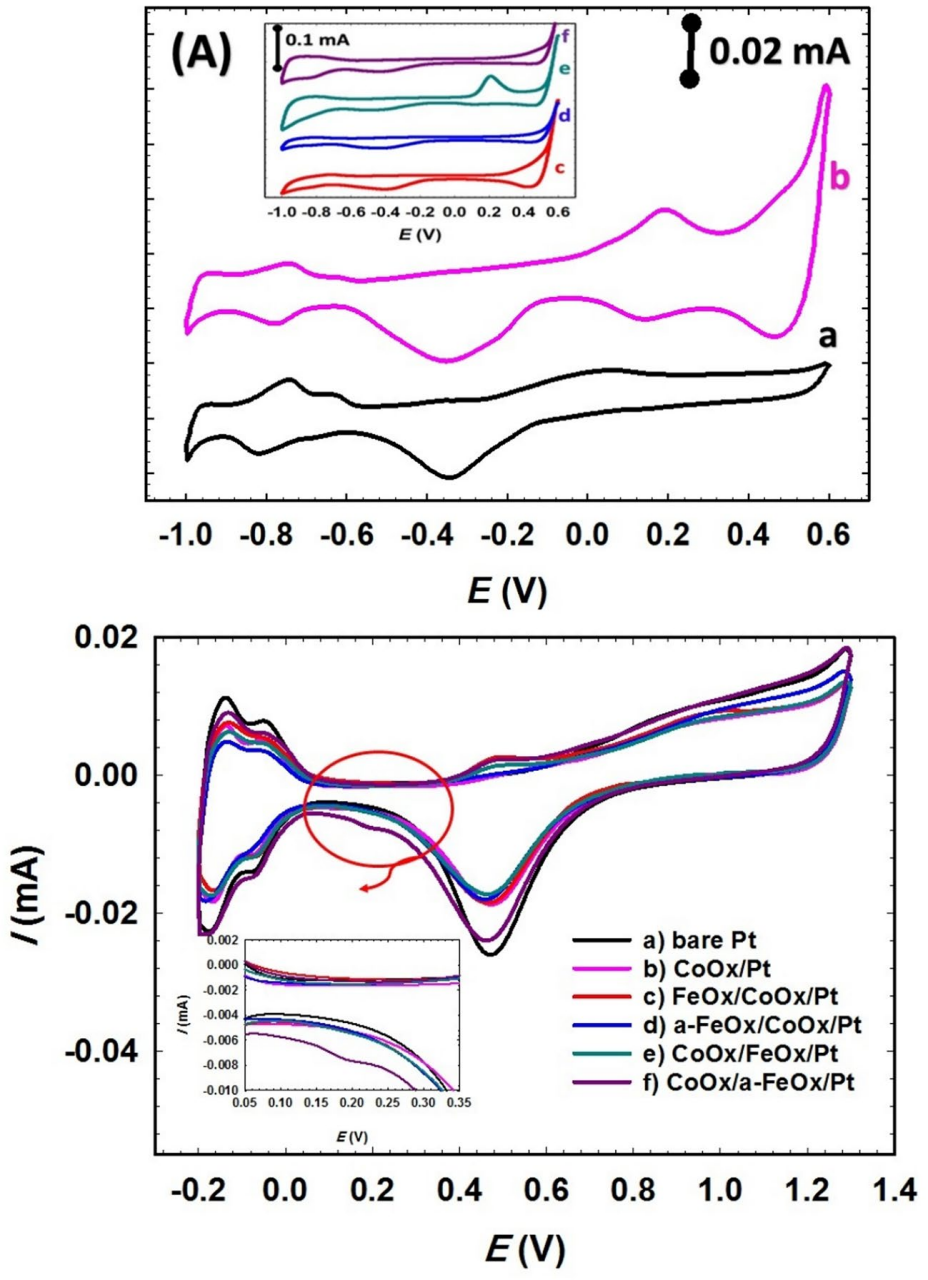
CVs obtained in 0.5 mol L^−1^ NaOH (A) and in 0.5 mol L^−1^ H_2_SO_4_ (B) for the (**a**) bare-Pt, (**b**) CoOx/Pt, (**c**) FeOx/CoOx/Pt, (**d**) a-FeOx/CoOx/Pt, (**e**) CoOx/FeOx/Pt and (**f**) CoOx/a-FeOx/Pt catalysts at a scan rate of 200 mV s^–1^.

The current in the electrocatalytic activity, *i-t* and CO stripping measurements was normalized to the electrochemical surface area (ECSA) that was calculated from the H_ads/des_ peaks (Fig. 1B).

### Preparation of catalysts

The simple electrodeposition method was utilized to fabricate the proposed catalysts. Prior to the electrodeposition process, the bare Pt electrode was mechanically polished with aqueous slurries of successively finer alumina powder, then re-polished with a 2500 grit emery paper to achieve a smooth surface before rinsing with distilled water. After that, in 0.5 mol L^−1^ H_2_SO_4_ solution, the Pt electrode was electrochemically pretreated by cycling the potential between − 0.2 and 1.3 V at 100 mV s^−1^ until the characteristic CV of a clean Pt surface was achieved^53,56,62^. The electrodeposition of nano-CoOx was performed by cycling the potential between 1.2 and − 1.1 V in an aqueous solution of 0.1 mol L^−1^ Na_2_SO_4_ containing 1.0 mmol L^−1^ CoSO_4_ at a scan rate of 100 mV s^−1^ (30 cycles of CoOx)^51^. Whereas the electrodeposition of nano-FeOx was performed by potential cycling between − 0.855 and − 1.205 V at a scan rate of 100 mV s^−1^ in 0.02 mol L^−1^ FeSO_4_.7H_2_O solution (2 cycles, FeOx)^56,58,63^. The activation of the nano-FeOx was carried out at a constant potential of − 0.5 V for 10 min in 0.2 mol L^−1^ NaOH aqueous solution. The catalyst name will reflect the sequence of the deposited layer, while the prefix “a-“ will indicates the post-activation of the catalyst after preparation.

## Results and discussion

### Electrochemical characterization

Characterizations of the as-prepared catalysts were studied electrochemically, and useful information regarding the catalytic ingredients was obtained. The electrochemical properties of the proposed catalysts were investigated in alkaline and acidic solutions. On one hand, Fig. 1A shows the CVs for the electrochemical response of the bare-Pt, CoOx/Pt, FeOx/CoOx/Pt, a-FeOx/CoOx/Pt, CoOx/FeOx/Pt and CoOx/a-FeOx/Pt catalysts in 0.5 mol L^−1^ NaOH at a scan rate of 200 mV s^−1^. In Fig. 1A (curve a), the distinctive behavior of polycrystalline Pt electrode was clearly seen; the oxidation of Pt, which extended over a wide range of potentials, the subsequent PtO reduction at ca. –0.35 V and the hydrogen adsorption/desorption (H_ads/des_) peaks at ca. –0.8 V^64^. After the modification of bare-Pt with nano-CoOx (CoOx/Pt catalyst, (curve b)), two distinct redox couples at ca. 0.2 and 0.5 V related to the transformation between nano-CoOx phases^65,66^. By further modification with nano-FeOx (FeOx/CoOx/Pt catalyst, (curve c)), small peaks assigned for nano-FeOx transformations were observed at ca. − 0.70 V (anodic peak) and ca. − 0.76 V (cathodic peak)^67^ together with the redox couples of nano-CoOx at 0.5 V, whereas the nano-CoOx transformation peaks at ca. 0.2 V vanished. It is noted that the peaks of nano-FeOx transformation were no longer observed after activation at − 0.5 V (curve d) which might result from the detachment of nano-FeOx to the solution during activation (later, this issue will deeply be addressed). However, when the deposition order is reversed, i.e., CoOx/FeOx/Pt catalyst (curve e), the nano-CoOx transformation peaks were obvious, in contrast to the peaks of nano-FeOx transformations^68^. Whereas, at the CoOx/a-FeOx/Pt catalyst (curve f), the nano-CoOx oxidation peaks at ca. 0.2 V faded away, leaving the other redox pair at 0.5 V. It seems during the activation of the CoOx/a-FeOx/Pt catalyst, nano-FeOx were dissolved and perhaps redeposited again on one of the nano-CoOx phases.

On the other hand, in a 0.5 mol L^–1^ H_2_SO_4_ solution (Fig. 1B) and at the surface of all catalysts, two peaks were obviously seen in the potential range of − 0.2 to 0.05 V for the H_ads/des_. The electrochemical surface area (ECSA) was calculated from the H_des_ peaks (Fig. 1B), according to this equation $ECSA \left({\text{cm}}^{2}\right)= \frac{{Q}_{H}(\mu C)}{210 (\mu C {\text{cm}}^{-2})}$, Q_H_ (µC) is the amount of hydrogen desorbed from the Pt surface and was calculated by integrating the area under the H_des_ peaks in the voltage range between –0.2 and 0.05 V. The value “210 µC cm^–2^” represents the typical charge consumed to desorb a monolayer of hydrogen atom from a Pt surface^62,63^. The anodic peak at ~ 0.48 V and the subsequent reduction at ~ 0.45 V were assigned to the Fe^2+^/Fe^3+^ conversions in nano-FeOx^56^. The reduction peak of this Fe^2+^/Fe^3+^ redox pair did not appear at all catalysts perhaps due to the stability of the high oxidation state of iron or probably due to its intensive dissolution in acidic media. The Pt oxidation at 0.7–1.2 V and the PtO reduction at ca. 0.48 V were also essential features for all catalysts. The redox peaks of nano-CoOx did not appear in H_2_SO_4_ solution because of its instability in highly acidic conditions^59^. One of the important features in Fig. 1B is the progressive decreases in ECSA with the steps of building up the a-FeOx/CoOx/Pt catalyst (Table 1), which interpreted the successful coverage of the nano-CoOx and nano-FeOx layers on top of the Pt surface. The activation step inspired a further decrease in the ECSA of the catalyst, which inferred possibly the oxidation of low iron states on the Pt surface. On contrast, the activation of the CoOx/a-FeOx/Pt catalyst inspired possibly the conversion of nano-FeOx early to a higher oxidation state of iron (note that the redox pair of Fe^2+^/Fe^3+^ conversions is the most obvious, see the inset of Fig. 1B) which enhanced the H_2_ spillover and, hence, the ECSA increased. The H_2_ spillover refers to the catalytic dissociation of H_2_ followed by its migration and diffusion on the surface and/or bulk catalyst^69^. Similar spillover was noticed previously for CO as well onto the a-FeOx/Pt catalyst^21^. On the other hand, and referring to the deposition of nano-CoOx onto the a-FeOx/Pt substrate (CoOx/a-FeOx/Pt catalyst) in the potential window between 1.2 and − 1.1 V, which can possibly alter the effect of activation of nano-FeOx, the increase of ECSA can be expected.

**Table 1:** Catalytic parameters ($ECSA, {I}_{p}^{d}$, ${I}_{p}^{ind}$, ${I}_{b}$, ${I}_{p}^{d}/{I}_{p}^{ind}$, ${I}_{p}^{d}/{I}_{b}$ and ${E}_{onset}$) of FAO as assessed from the data of Fig. 5**.**

Electrode	ECSA (cm^2^)	${I}_{p}^{d}$(mA cm^−2^)	${I}_{p}^{ind}$(mA cm^−2^)	${I}_{b}$(mA cm^−2^)	${I}_{p}^{d}/{I}_{p}^{ind}$	${I}_{p}^{d}/{I}_{b}$	${E}_{onset}$(mV)
bare-Pt	0.010	4.5	2.0	14.5	2.3	0.31	− 74
CoOx/Pt	0.0070	8.5	3.2	16.5	2.7	0.52	− 70
FeOx/CoOx/Pt	0.0064	17.9	1.7	23.2	10.5	0.77	− 106
a-FeOx/CoOx/Pt	0.0056	16.4	1.7	23.3	9.6	0.70	− 114
CoOx/FeOx/Pt	0.0066	14.8	1.5	22.8	9.9	0.65	− 60
CoOx/a-FeOx/Pt	0.0089	10.9	1.2	16.3	9.1	0.67	− 32

**Figure 2:**
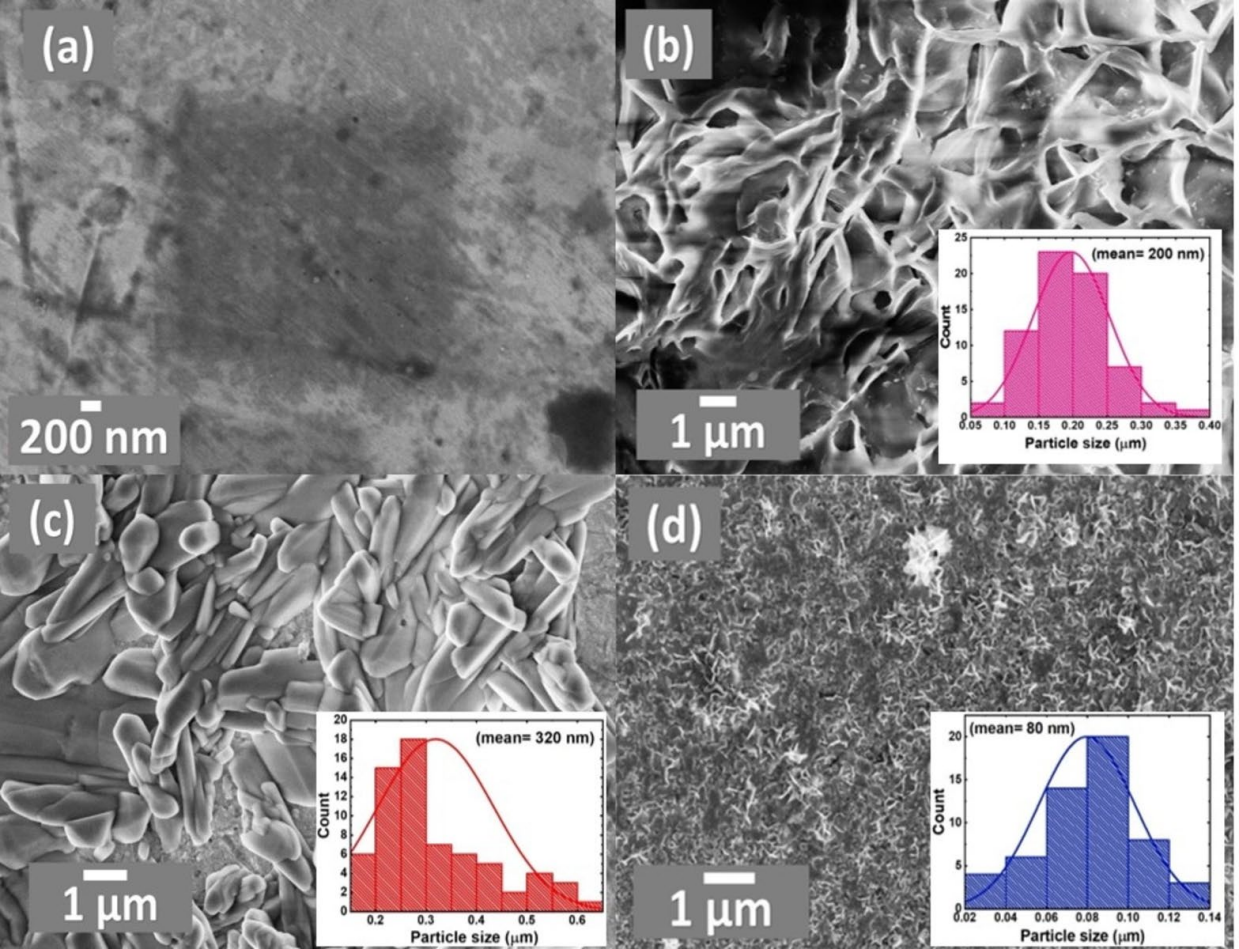
FE-SEM micrographs obtained for the (**a**) bare-Pt, (**b**) CoOx/Pt, (**c**) FeOx/CoOx/Pt and (**d**) a-FeOx/CoOx/Pt catalysts.

**Figure 3:**
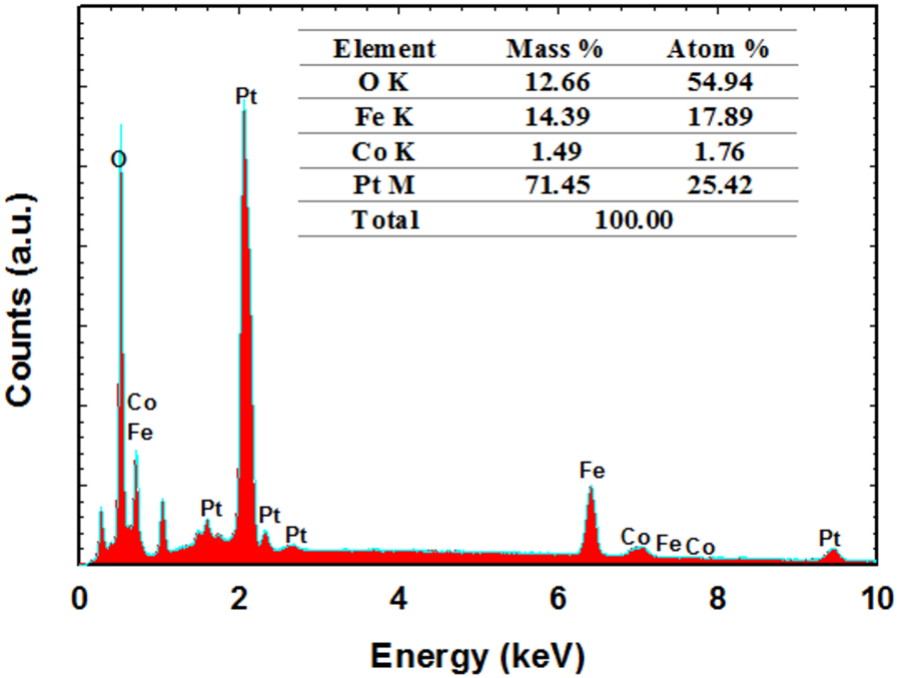
The EDX spectrum of the FeOx/CoOx/Pt catalyst.

Geometrically, the FE-SEM in Fig. 2 assisted in evaluating the morphology of the proposed catalysts. The bare-Pt surface appeared featureless in Fig. 2a. However, nano-CoOx was deposited onto the bare Pt surface in the form of a porous network of interconnected fibers and sheets (200 nm in a particle size), as seen in Fig. 2b. On the other hand, nano-FeOx was deposited onto the CoOx/Pt catalyst in large flakes and rod-like texture of lengths in the range of 1–5 μm and a particle size of 320 nm (see Fig. 2c). Finally, the activation of nano-FeOx in the a-FeOx/CoOx/Pt catalyst sustained nano-FeOx in a much thinner and shorter nanorods of 80 nm in a particle size and 253 nm in average length, (Fig. 2d). The chemical composition of this FeOx/CoOx/Pt catalyst was determined by the EDX analysis (Fig. 3) which confirmed the successful deposition of the different ingredients (nano-CoOx and nano-FeOx) onto the bare-Pt surface and assisted in predicting their relative ratios (inset of Fig. 3).

**Figure 4:**
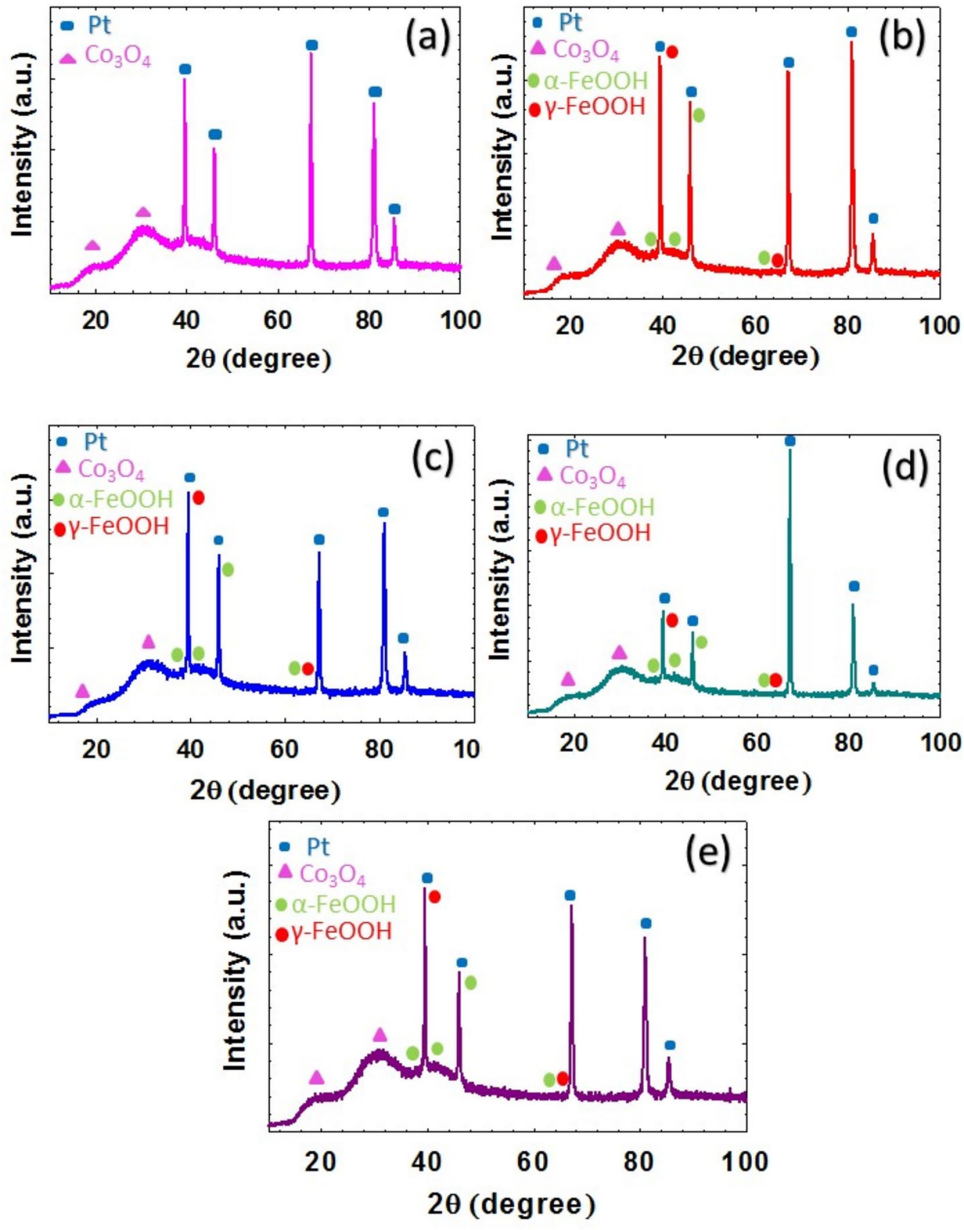
XRD patterns of (**a**) CoOx/Pt, (**b**) FeOx/CoOx/Pt, (**c**) a-FeOx/CoOx/Pt, (**d**) CoOx/FeOx/Pt and (**e**) CoOx/a-FeOx/Pt catalysts.

The crystal structures of the CoOx/Pt, FeOx/CoOx/Pt, a-FeOx/CoOx/Pt, CoOx/FeOx/Pt, and CoOx/a-FeOx/Pt catalysts were analyzed using XRD (Fig. 4). The typical diffraction peaks of the face-centered cubic (FCC) of Pt at *2θ* 39.5°, 46.0°, 67.2°, 81.0° and 85.5° were obviously seen for all catalysts. These belonged, respectively, to the (1 1 1), (2 0 0), (2 2 0), (3 1 1) and (2 2 2) facets of the FCC Pt (JCPDS card no. 00-004-0802)^56^. The high intensities of the Pt diffractions in Fig. 4 were consistent with the nature of the bulk Pt substrate. Moreover, all catalysts in Fig. 4 retained diffraction peaks at ca. 19.5° and 32°, which corresponded to the (1 1 1) and (2 2 0) spinel phase of Co_3_O_4_ (JCPDS card no. 43-1003)^21,70^. On the other hand, the diffraction peaks at 37°, 42.1°, 46.3° and 62.3° were assigned to the (1 1 1), (1 4 0), (0 4 1) and (0 0 2) planes of α-FeOOH (JCPDS card no. 29–0713). Additional diffraction peaks at ca. 40° and 63° were attributed to the (1 0 1) and (1 1 0) planes of γ-FeOOH (JCPDS card no.13-0087)^57^.

### FAO: catalytic activity

**Figure 5:**
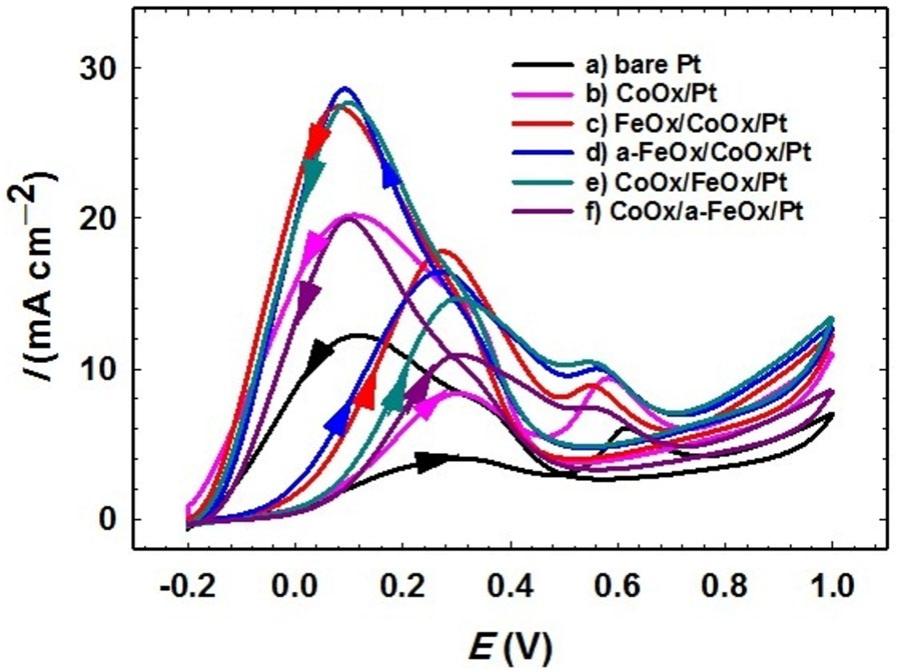
FAO at the (**a**) bare-Pt, (**b**) CoOx/Pt, (**c**) FeOx/CoOx/Pt, (**d**) a-FeOx/CoOx/Pt, (**e**) CoOx/FeOx/Pt and (**f**) CoOx/a-FeOx/Pt catalysts in 0.3 mol L^–1^ FA (pH 3.5) at a scan rate of 100 mV s^–1^.

The electrocatalysis of FAO at the CoOx/Pt, FeOx/CoOx/Pt, a-FeOx/CoOx/Pt, CoOx/FeOx/Pt and CoOx/a-FeOx/Pt catalysts was inspected and compared to that at the bare-Pt in 0.3 mol L^−1^ HCOOH (pH = 3.5) solution. First of all, we should emphasize the inactivity of the nano-CoOx and nano-FeOx to FAO, as previously reported^56^. Generally, for all catalysts, as long as there is an access for Pt to the electrolyte, three peaks are noticed (Fig. 5); the first is anodic at ca. 0.3 V for the direct dehydrogenation pathway of FAO where its peak current ($${I}_{p}^{d})$$ reflects this pathway's preference. The second is also anodic but observed at a higher overpotential (ca. 0.65 V) and is related to the indirect dehydration pathway of FAO, in which FA is dehydrated at open circuit voltage; releasing poisonous CO (CO_ads_) that gets adsorbed strongly at the Pt surface; competing the FA adsorption and deactivating the direct pathway of FAO. At high overpotentials (at ca. 0.65 V), when the Pt surface becomes enriched with –OH groups, CO_ads_ is oxidized where the peak current ($${I}_{p}^{ind})$$ depicts the poisoning level. The third peak is in the backward “cathodic-ongoing” direction and has a peak current ($${I}_{b})$$ corresponding to several faradaic processes; including the direct FAO on a cleaned “non-poisoned” Pt surface, reduction of CO_2_ and Pt surface dehydroxylation^71^. Looking deeply to Fig. 5 indicates the increase of $${I}_{p}^{d}$$ in the following order: (bare-Pt < CoOx/Pt < CoOx/a-FeOx/Pt < CoOx/FeOx/Pt < a-FeOx/CoOx/Pt < FeOx/CoOx/Pt). This recommends a more favourable FAO in the direct pathway in the same order. This magnitude of $${I}_{p}^{d}$$, is, indeed, one of the elements that we considered in assessing the catalytic activity. Another element considered the relative values of $${I}_{p}^{ind}$$, and herein, all catalysts (except CoOx/Pt) retained lower values than that of the bare-Pt, which highlighted again their favourable potential to mitgate the CO poisoning. Moreover, all the catalysts depicted higher $${I}_{b}$$ values (a third element of comparison) than that of the bare-Pt electrode, which was consistent with lower CO_ads_ poisoning levels and enhanced FAO. From another perspective, a comparison of the $${I}_{p}^{d}$$/$${I}_{p}^{ind}$$ and $${I}_{p}^{d}$$/$${I}_{b}$$ ratios of the different catalysts are able to correlate, respectively, their catalytic activity and catalytic tolerance against CO poisoning. A quick inspection of the data in Table 1 exalts mostly the catalytic perfomance of the FeOx/CoOx/Pt catalyst, in accordance to its honor in terms of $${I}_{p}^{d}$$. It mainained $${I}_{p}^{d}$$/$${I}_{p}^{ind}$$ and $${I}_{p}^{d}$$/$${I}_{b}$$ ratios of 10.5 and 0.77, respectively, that were almost 4.6 and 2.5 times that of the corresponding values obtained at the bare Pt catalyst, with ca. − 106 V shift in the onset potential, $${E}_{onset}$$. The existence of nano-CoOx as an intermediate layer in the FeOx/CoOx/Pt catalyst boosted largely its catalytic performance toward FAO. Comparing the respective $${I}_{p}^{d}$$, $${I}_{p}^{d}/{I}_{p}^{ind}$$ and $${I}_{p}^{d}/{I}_{b}$$ of FAO at the FeOx/Pt (ca. 6.4 mA cm^–2^, 9.1 and 0.58) and FeOx/CoOx/Pt (ca. 18 mA cm^–2^, 10.5 and 0.77) catalysts was enough to highlight this role^56^. Hence, in terms of activity, the FeOx/CoOx/Pt catalyst was quantitatively the most efficient not only among the catalysts of this study but also in comparison to literature data, Table 2^50,54,56,57,72–77^.

**Table 2:** A comparison of the activity of the inspected catalysts with literature data for FAO.

Catalyst	${I}_{p}^{d}$(mA cm^−2^)	${I}_{p}^{d}/{I}_{p}^{ind}$	${I}_{p}^{d}/{I}_{b}$	Refs.
Pt_11.1_Ni_88.9_/C	–	0.33	–	^72^
Pt_10.9_Au_0.2_Ni_88_._9_/C	–	0.34	–	^72^
Mn/Pt/GC	–	3.13	0.50	^54^
NiOx/Pt/GC	–	3.33	0.40	^50^
Pt_1_Cu_4_/GC	–	3.58	0.73	^73^
PtPd/GC	–	7.33	0.32	^77^
Pt/MWCNTs-GC	–	7.5	0.45	^74^
FeOx/Pt	6.4	9.1	0.58	^56^
a-FeOx/Pt	8.7	17.4	0.70	^56^
Si-TiOx/Pt/TiOx (700 °C)	–	10.0	0.67	^75^
Au_23_/Pt_63_Co_14_ HNWs/C	11.7	3.7	–	^76^
FeOOH/Pt/GC	15.0	5.0	0.35	^57^
bare-Pt	4.5	2.3	0.31	This Work
FeOx/CoOx/Pt	17.9	10.5	0.77	This Work

### FAO: stability testing

**Figure 6:**
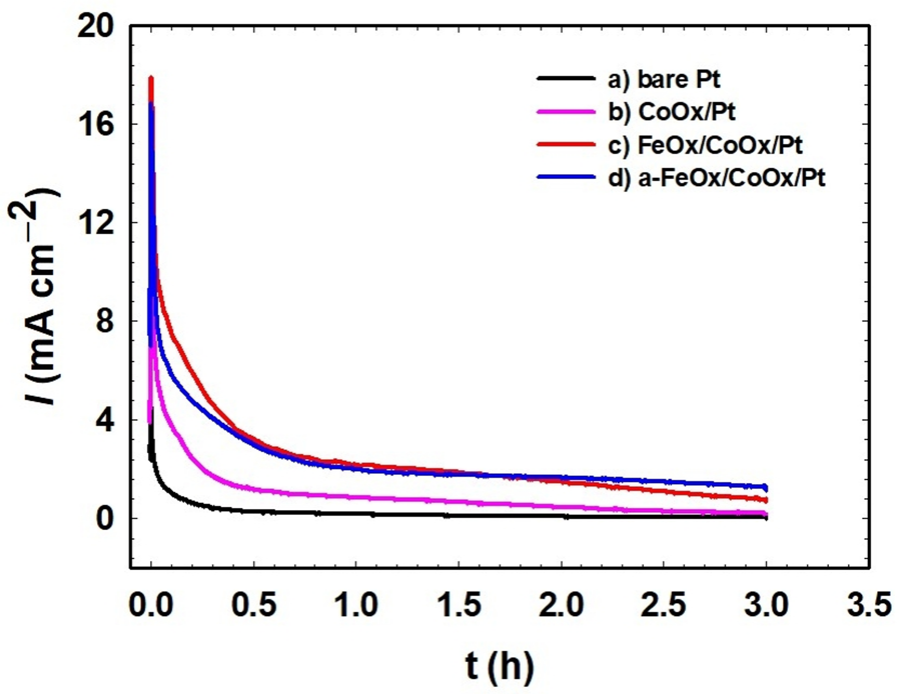
CA of the (**a**) bare-Pt, (**b**) CoOx/Pt, (**c**) FeOx/CoOx/Pt and (**d**) a-FeOx/CoOx/Pt catalysts in 0.3 mol L^−1^ FA (pH 3.5) during FAO at 50 mV.

The electrochemical stability of the proposed catalysts was investigated via chroamperometric (CA) measurements in which the (*i–t*) relations in 0.3 mol L^–1^ FA, pH = 3.5 at a constant applied potential of 50 mV for 3 h were plotted. Figure 6a–d apparently shows a fast decay in the current densities after few minutes of electrolysis which is attributed to the accumulation of poisonous CO and CO-like intermediates at the catalyst surface^78^. However, after 3 h of continuous electrolysis, the a-FeOOH/CoOx/Pt (Fig. 6d) and FeOx/CoOx/Pt (Fig. 6c) catalysts exhibited, respectively, ca. 15 and 10 times higher current densities than that obtained at the bare-Pt (Fig. 6a) catalyst; indicating better resistances for poisoning with CO and CO-like intermediates, in consistent with the data obtained in Fig. 5. The CoOx/Pt catalyst (Fig. 6b) approached the bare-Pt current after 3 h of continous electrolysis. This inspection valued the role of catalyst's activation in improving the catalyst's stability, which is highly important for the industrial scales (note the stability of current after 1 h in Fig. 6d).

### EIS measurements

**Figure 7:**
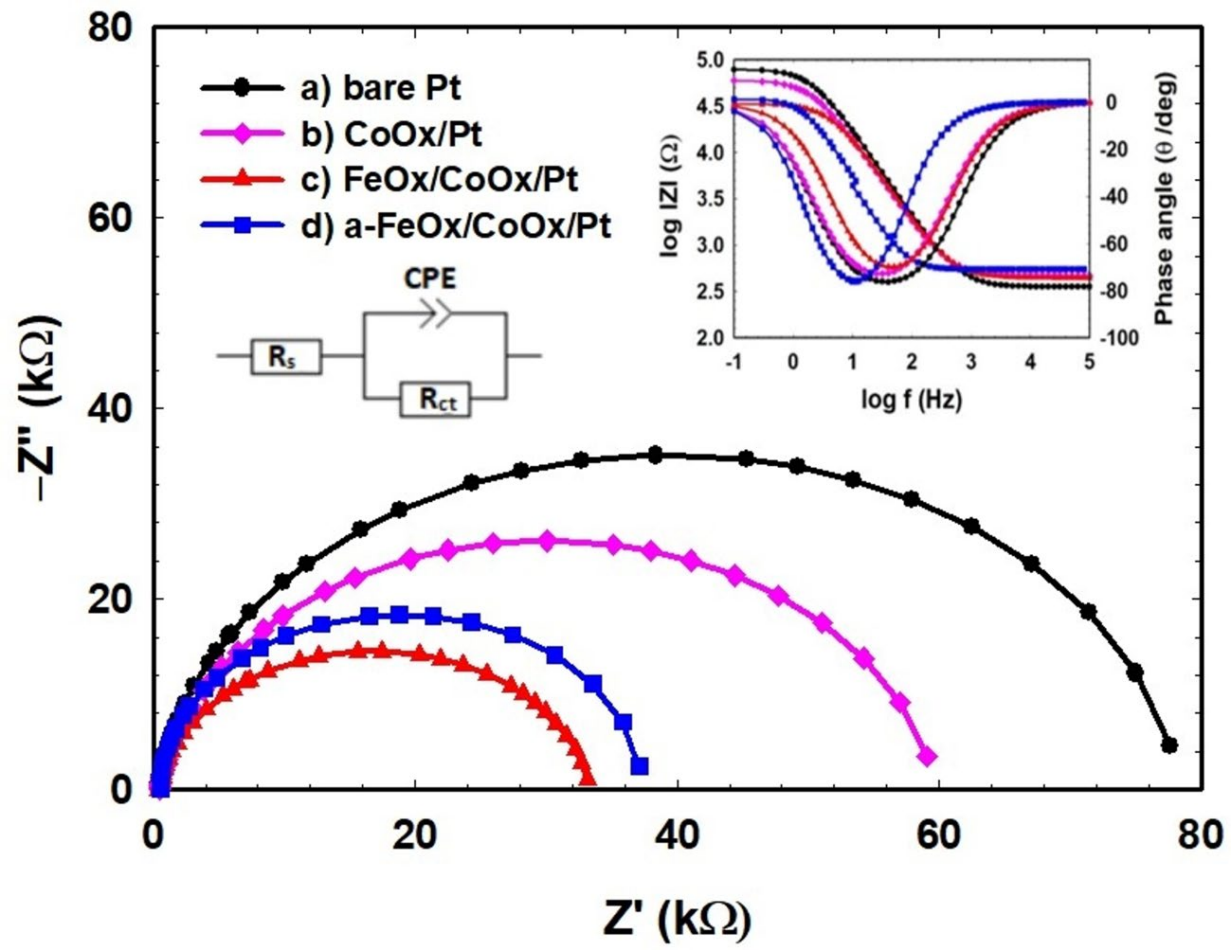
Nyquist and Bode plots (at open circuit potentials) for the (**a**) bare-Pt, (**b**) CoOx/Pt, (c) FeOx/CoOx/Pt and (d) a-FeOx/CoOx/Pt (activated at − 0.5 V for 10 min) catalysts in 0.3 mol L^−1^ FA (pH 3.5).

The electrochemical impedance spectroscopy (EIS) was used to estimate the charge transfer resistance (R_ct_) of the prepared catalysts in FAO. Nyquist and Bode plots were measured at open circuit potentials in an aqueous solution of 0.3 mol L^−1^ FA (pH = 3.5) in the frequency range (10 mHz–100 kHz), see Fig. 7. Nyquist plot represented semi-circles which confirmed a kinetically controlled reaction^79^. The experimentally measured EIS data were fitted using Randle’s equivalent circuit and the corresponding equivalent circuit of this system is shown in the inset of Fig. 7. Table 3 lists the impedance parameters as R_ct_, Ohmic resistance (R_s_) and constant phase element (CPE) of the various catalysts. The R_ct_ values clearly declined in the order bare-Pt (78.1 kΩ) > CoOx/Pt (59.3 kΩ) > a-FeOx/CoOx/Pt (36.7 kΩ) > FeOx/CoOx/Pt (32.9 kΩ). This evidence helped in understanding the improved electrocatalytic activity of the FeOx/CoOx/Pt and a-FeOx/CoOx/Pt catalysts toward FAO, which most likely was originated electronically.

**Table 3:** EIS parameters obtained Fig. 7 for FAO at various catalysts.

Electrode	R_s_ [kΩ]	R_ct_ [kΩ]	CPE [µFs^(n−1)^]	n
bare-Pt	0.354	78.1	1.189	0.9323
CoOx/Pt	0.467	59.3	1.570	0.9200
FeOx/CoOx/Pt	0.444	32.9	1.466	0.9208
a-FeOx/CoOx/Pt	0.548	36.7	2.909	1.0

### CO stripping measurements

**Figure 8:**
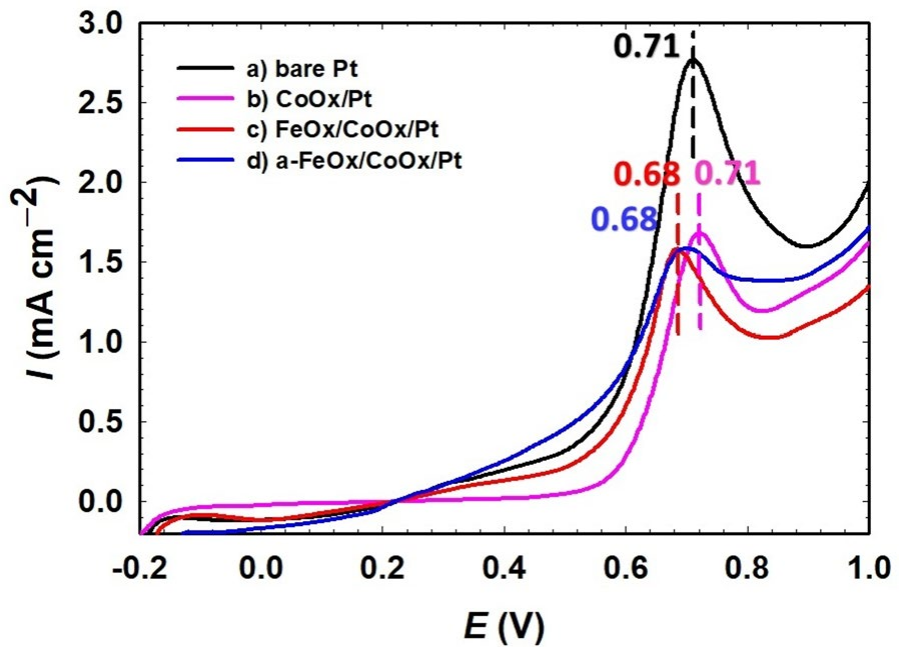
Oxidative CO stripping at the (**a**) bare-Pt, (**b**) CoOx/Pt, (**c**) FeOx/CoOx/Pt and (**d**) a-FeOx/CoOx/Pt catalysts in 0.5 mol L^−1^ H_2_SO_4_ measured at 50 mV s^–1^.

The adsorption of poisoning intermediate (CO_ads_) during FAO can definitely hinder the safe arrival of FA flux to the Pt surface, reducing the oxidation efficiency^80^. A CO-stripping experiment was performed to investigate the catalysts' tolerance for CO_ads_^81^. To understand the roles of nano-FeOx and nano-CoOx in the catalytic enhancement of FAO, CO was chemisorbed from 0.5 mol L^−1^ FA at an open circuit potential at the proposed catalysts for 10 min^21^. After that, this adsorbed CO layer was stripped electrochemically in 0.5 mol L^−1^ H_2_SO_4_, as illustrated in Fig. 8. The CO electro-oxidation peak in Fig. 8 was recorded for the first cycle of CO stripping. The catalysts' onset potentials were arranged as following: a-FeOx/CoOx/Pt (0.30 V) < FeOx/CoOx/Pt (0.45 V) < CoOx/Pt (0.50 V) < bare Pt (0.50 V). The a-FeOx/CoOx/Pt catalyst exhibited the lowest onset potential among the whole set of catalysts which indicated the favorable engineering of its surface to facilitate the CO desorption. The existence of nano-FeOx and nano-CoOx in this hierarchy could presumably enrich the Pt surface with the hydroxyl groups required to initiate the CO desorption. The existence of these oxides could moreover amend the surface electronic structure of Pt in the way weakening the Pt–CO bonding. The highest CO oxidation peak current (2.8 mA cm^–2^ at 0.71 V) was obtained at the Pt electrode, indicating its subjection to the highest poisoning level with CO_ads_. The CO oxidation peaks at the CoOx/Pt, FeOx/CoOx/Pt and a-FeOx/CoOx/Pt catalysts appeared at 0.71, 0.68 and 0.68 V, with peak current densities of ca. 1.7, 1.5 and 1.5 mA cm^−2^, respectively. It means that all the modified catalysts; CoOx/Pt, FeOx/CoOx/Pt and a-FeOx/CoOx/Pt, enjoyed much better mitigation of CO adsorption and that resulted geometrically or bifunctionally (as revealed from the lower charged that was consumed in the CO stripping peak) and/or electronically (from the − ve shift in the peak and onset potentials). It is worth pointing that shifting ${E}_{onset}$ of CO stripping in the negative potential direction correlates to weaker CO adsorption at the catalyst. These bifunctional and electronic effects can possibly consort the accommodation of electrons involved in FAO in the vacant d-orbitals of Fe and Co in the way speeding the oxidation kinetics.

### The reaction mechanism

As indicated before, the direct pathway (dehydrogenation) mechanism of FAO dominates at low applied potentials, resulting in CO_2_ formation. This dehydrogenation pathway involves the formation/disappearance of formate (HCOO^–^) intermediate. The released protons may affect the surface of TMOs (nano-CoOx and/or nano-FeOx), modulating significantly their catalytic activity^82^. Hence, a plausible justification for the synergetic catalytic enhancement of FAO in presence of nano-CoOx and nano-FeOx nanoparticles can adopt the following reversible transformation between the lower (II, $\text{H}-\text{O}-\text{M}$) and higher (III, $\text{O}-\text{M}$) oxidation states of Co and Fe in their oxides.

1 $${\text{Co}(\text{OH})}_{2}\leftrightarrow \text{CoOOH}+{\text{H}}^{+}+{\text{e}}^{-}$$

2 $${\text{Fe}(\text{OH})}_{2}\leftrightarrow \text{FeOOH}+{\text{H}}^{+}+{\text{e}}^{-}$$

Accordingly, the CoOOH and/or FeOOH (represented as $\text{O}-\text{M}$) can mediate the mechanism of FAO according to these equations:

3 $$\text{HCOOH}+\text{O}-\text{M}{ +\text{ e}}^{-}\to {\text{HCOO}}^{-}+\text{H}-\text{O}-\text{M}$$

4 $${\text{HCOO}}^{-}+\text{H}-\text{O}-\text{M}\to {\text{O}-\text{M}+\text{CO}}_{2}+2{\text{H}}^{+}+{3\text{e}}^{-}$$

The overall reaction is, therefore:

5 $$\text{HCOOH}\to {\text{CO}}_{2}+{2\text{H}}^{+}{ + 2\text{e}}^{-}$$

## Conclusion

A novel ternary catalyst composed of nano-CoOx and nano-FeOx that were electrodeposited sequentially onto a bare Pt electrode was recommended for efficient formic acid electro-oxidation (FAO). The deposition order of the catalytic ingredients (i.e., nano-CoOx and nano-FeOx) of the catalyst influenced greatly the catalytic performance toward FAO. The highest activity was obtained at the FeOx/CoOx/Pt electrode (for which, the nano-CoOx was first deposited onto the Pt surface, followed by nano-FeOx). Compared to the bare Pt electrode, the FeOx/CoOx/Pt catalyst exhibited 4.6 times higher effeciency for FAO, with up to 2.5 increase in the catalytic tolerance against CO poisoning and to − 32 mV shift in ${E}_{onset}$. It also enjoyed much better durabilitity for long-termed electrolysis. The EIS and CO stripping voltammetry confirmed both geometrical and electronic contributions in the catalytic enhancement of FAO at the FeOx/CoOx/Pt catalyst.

## Data Availability

Data are provided within the manuscript.
